# Through the Lens of Psychiatrists: Understanding Smoking Cessation Behaviour Among People with Serious Mental Illness

**DOI:** 10.1007/s10597-025-01460-1

**Published:** 2025-02-27

**Authors:** Parul Parul, Bindu Joseph, Sunil Datta, Avinash Desousa, Muhammad Aziz Rahman

**Affiliations:** 1 Institute of Health and Wellbeing, Federation University Australia, Berwick, VIC 3806 Australia; 2 The Royal Melbourne Hospital, Melbourne, Australia; 3 De Sousa Foundation, Carmel 18, St Francis Ave, Willingdon, Santacruz West, Mumbai, Maharashtra 400054 India; 4 School of Nursing, Institute of Health and Management, North Melbourne, VIC Australia; 5 Faculty of Public Health, Universitas Airlangga, Surabaya, Indonesia

**Keywords:** Psychiatrists, Serious mental illness, Smoking, Cessation, Qualitative

## Abstract

Smoking among people with Serious Mental Illness (SMI) is a pleading public health concern as the rates are much higher compared to the general population. Although different interventions are available to provide cessation support, there is limited evidence of assessing psychiatrists’ perspectives on smoking cessation among people with SMI. The study aims to explore the perspectives of psychiatrists regarding smoking cessation among people with SMI. The study used a qualitative approach. In-depth interviews were conducted with ten psychiatrists, exploring their opinions about smoking and its cessation among people with SMI. Purposive sampling was employed. Data saturation was achieved when no new information was gathered. The verbatim transcripts were analysed using thematic analyses. A total of 14 subthemes emerged under seven themes, highlighting the perspective of psychiatrists in terms of the utilisation of smoking as a coping mechanism among people with SMI. Findings, such as priority of care, time constraints and patient initiative, emphasised the factors impacting cessation. Psychiatrists expressed that support from peers and family members significantly influences smoking cessation among people with SMI. Furthermore, merely engaging in asking, assessing and advicing components from the World Health Organisation 5As scale implied insufficient usage of the 5As tool while discussing cessation. Psychiatrists recognise the impact of cessation on mental health outcomes, like the utilisation of smoking as a coping mechanism. Their experiences and challenges with cessation highlighted time commitment and priority of care while contemplating cessation. The deepened insight provided by our study findings has been instrumental in shaping the content of tailored interventions related to smoking cessation among people with SMI.

## Introduction

Smoking is the world’s leading cause of preventable illness and death (World Health Organization, 2023). Globally, the prevalence of smoking has decreased from 29 to 15% in recent years (Reitsma et al., 2017). However, people with mental health issues have been more likely to smoke compared to the general population (Lê Cook et al., 2014). The rate of tobacco screening and treatment in mental health care was low (Rogers & Sherman, 2014). A significant barrier was misunderstanding of the role of smoking in mental health issues, which led to difficulties in quitting and ultimately impacting treatment (Sheals et al., 2016). SMI refers to a mental, behavioural, or emotional disorder resulting in serious functional impairment, which substantially interferes with or limits one or more major life activities (National Institute of Mental Health, n.d.). It is well-proven that quitting smoking leads to improvement in mental health, and there were no discrepancies between continuing combined treatment for mental health issues and smoking cessation (Taylor et al., 2020).

Mental health practitioners play a crucial role in triggering quit attempts among patients who want to quit (Stead et al., 2013); still, psychiatrists held some common beliefs in terms of low self-efficacy, ineffective cessation support, and time constraints as some of the identified barriers (Twyman et al., 2014). Another significant hindrance identified was the lack of attention of practitioners in terms of priority towards smoking cessation (Pagano et al., 2016). A recent systematic review confirmed that mental health professionals had negative attitudes related to smoking cessation (with sub-themes: Patients are not interested in quitting, cessation is not a priority and is not part of their role) for people with mental health issues (Sheals et al., 2016). A combination of drugs and behavioural therapies has been effective for cessation, yet it was minimally applied in quit attempts (Stead et al., 2016).

Providers’ knowledge and views on cessation need to be integrated into planning cessation (Taylor et al., 2021). It has become obligatory to remind providers regarding smoking screening in the first instance and refer patients to appropriate specialists/counsellors for resolving smoking dependence so that the current priorities in care and busy workflow would not hinder while talking about cessation (Rogers et al., 2018). People with mental health issues choose to get support from their psychiatrists related to quitting instead of other health professionals (Malone et al., 2018). Patients with an existing smoking-related illness were more inclined toward working on the proposed plan to quit smoking (Malone et al., 2018; Papadakis et al., 2020). There is a need to explore opportunities for smoking cessation while assisting in recovery from mental health issues, which emerge for elaborative understanding so the best evidence-based interventions can be implemented successfully. Most of the available research examined various factors that impede smoking cessation for people with SMI and has been conducted through quantitative methods with predictive factors. The current study focuses on exploring the perspectives of psychiatrists related to smoking cessation among people with SMI.

## Methods

### Study Design

The study utilised a qualitative exploratory, descriptive approach, which allows for the exploration, description, and provision of the phenomenon of interest that moves beyond mere observation (Hunter et al., 2019). This study involved conducting one-to-one online in-depth interviews with psychiatrists about smoking cessation among people with SMI.

### Participants and Settings

The participants were psychiatrists (n = 10) currently practising in psychiatric clinical settings in India who were enrolled in the Indian Association of Private Psychiatry [IAPP] (IAPP, n.d.). At the end of the cross-sectional survey, participants were asked about their willingness to participate in further interviews. Based on the consent, participants were approached considering varied demographics and other factors (such as age, gender, and clinical experience). Purposeful sampling was used to recruit participants.

### Procedure for the Interviews

An interview guide, after a thorough review of the literature and identification of gaps, was developed. Researchers conducted online in-depth interviews in October 2023 with pre-planned invitations sent to all participants through the Microsoft Teams application, considering the participants’ availability. All the interviews were audio–video recorded and conducted in English. The interviews were recorded and transcribed verbatim, guided by the objectives and principles of Kalu and Bwalya (2017), and stored. The recruitment goal for the present study was ten participants. Data saturation was achieved in seven interviews, indicating redundancy (Braun & Clarke, 2021); still, ten interviews were conducted to validate and confirm the accuracy of the information.

### Ethical Considerations

Ethics approval was obtained from the Human Research Ethics Committee (HREC) of Federation University Australia (Project number: 2023/006) to conduct this study. The Plain Language Information Statement (PLIS) was discussed before starting the interview. Participants’ responses were de-identified entirely to ensure anonymity. The explanatory statement fully informed participants about the research project, and they had the right to withdraw at any time during the research process. Verbal consent was obtained through recorded online interviews. Data collected from the interview guide and transcribed data were securely stored in the researcher’s password-protected university OneDrive cloud.

### Data Analysis

Following six phases of Braun and Clarke (2006) framework of data analysis, thematic analysis commenced promptly after the interview transcription, with multiple readings to grasp the meaning and foster familiarity with the contents. Later, through manual coding, a profound comprehension and deeper insight into the data were attained. The first step involved note-taking and organising the data according to codes, achieving data familiarisation. In the next step, initial codes emerged through manual dataset coding based on the research questions. These codes were then gathered and grouped based on their meanings, constituting initial themes in step four. In step five, themes were refined and renamed, and these were grouped according to the research question and relevant categories. A comprehensive analysis report, developed through discussions with co-researchers, ensured alignment with the study’s research question and existing literature. Consistency was enhanced through scrutiny by proficient researchers, resolving inconsistencies collaboratively.

## Results

### Participants Details

Ten psychiatrists were involved, with a mean age of 43, comprising six males and four females. Half of them had over 6 years of clinical experience in mental health settings. In terms of their work areas, four worked in both the government and private sectors (psychiatric hospitals), while three were employed exclusively in the government sector and three in the private sector.

### Themes and Subthemes

Table 1 provides an overview of themes and subthemes. Each theme and subtheme were addressed in detail later.

**Table 1:** Themes and Subthemes

Sr	Themes	Subthemes	Example Quotations
1	Smoking as a coping mechanism	–	*P 10: Truly speaking, it’s a multifaceted issue, and it differs from person to person. The majority of them use it as a coping mechanism*
2	The Consequences of smoking on mental health issues	Efficacy of psychometric medication	*P3: Smoking initiates problems in the metabolism of the drug so that the way patients get poorer responses with psychotropics prescribed by us*
		Smoking is a costly affair	*P2: Smoking cigarettes is a costly affair. This financial restraint leads to the stopping of medicines rather than stopping cigarettes. And then there is a relapse of illness, and that vicious cycle keeps on repeating*
3	Smoking cessation: Patient-related factors	External factors	*P3: Majorly the peer group or the easy availability of these substances or cigarettes makes them more indulged towards smoking, which actually forces them towards cessation*
		Internal factors	*P1: They are poorly motivated, even though they initiate smoking cessation, they may not be able to go through it*
4	Smoking cessation: Psychiatrist-related factors	Lack of time	*P6: Time is always the barrier because I mean, of course, my clients would like to spend two hours with me. But as far as I’m concerned. If I have those hours for my general OPD, and I try to see only a set number of patients*
		Priority of care	*P7: And lastly, I think our doctors’ perception was another problem, as most of them believe it is not a big problem*
		Lack of knowledge	*P8: Definitely, lack of training at the PG level among doctors can impact the cessation, which I think is a huge factor. I mean, I’ve trained so many years back, but we don’t get specific training for tobacco cessation*
		Patient initiative/ accountability	*P3: One of the things that we don’t try much. You have accepted that you are going to smoke. And I’m going to even if I break my head over it again multiple times, so most of us have accepted that. Yeah, it’s until the patient wants to stop smoking*
5	Catching the Craving: Applying the 5As	Consistently engaging in 3As (Asking, Assessing and Advicing)	*P9: Truly speaking, I think asking, advising, and assessing is easy on our part, as these are on the psychiatrist’s part. We utilise these components during the first patient visit*
		Challenges associated with the rest of 2As (Assisting and Arranging)	*P4: I can say assisting and arranging is a bit difficult for patients. Though I tried my best, I know sometimes. All of our efforts are not enough*
6	Experience and challenges around “cessation talk.”	Initiating cessation talk	*P6: When the symptoms are better, they’re doing well, they’re maintained on medications, and their functioning has improved a little bit. We have then those conversations. Then we know that the motivation isn’t that great, but we make a point, and then we ask again next month*
		Lack of readiness	*P5: The majority, as I told you, are not willing to stop, and they are not happy to abstain from their smoking activities. So they’re absolutely fine, and whatever other disorder, depression, bipolar, alcohol, they are willing to take the treatment for the same*
		Rolling with resistance	*P2: We focus on treating the present condition, like treating schizophrenia; in the next couple of visits, we will later target cessation because that is going to affect my treatment. It is their habit, so we can’t test the challenge directly; if we do so, he will not come to me for a follow-up of his psychiatric treatment*
		Highlighted physical health outcome	*P9: You see, most of our patients aren’t really eager to quit smoking unless they face serious health issues like a stroke or a heart attack caused by smoking. That’s when they become more motivated to quit to treat their physical illness, which they fear dying and asking to resolve the issue of smoking. That’s when we start talking about stopping smoking*
7	Decoding demographics and Recommendations	–	*P2: We have seen mainly the male gender, those who smoke a lot in early adulthood or late adolescence and the rural population*

### Theme 1: Smoking as a Coping Mechanism

The most common concept that emerged among participants was smoking as a “coping mechanism” in terms of their beliefs related to smoking among people with SMI.

1. *The first thing we see in a lot of patients who have SMI is that they use smoking as a coping mechanism. People with schizophrenia use smoking as a coping mechanism; it reduces their hallucinations and their thought processes.*

Many of them agreed with findings from scientific research confirming that smoking helps in resolving psychiatric symptoms. This justifies why people with SMI have repetitive usage of tobacco.

P8: *As scientific evidence is there, it confirms that because smoking activates nicotinic receptors, it also helps in reducing auditory hallucinations among patients with schizophrenia. So, they use that as a coping mechanism to take care of their hallucinations.*
P10: *Truly speaking, it’s a multifaceted issue, and it differs from person to person. The majority of them use it as a coping mechanism.*

Some of the participants revealed that stress is one of the crucial components for the inclination of smoking among people with SMI. Managing stress while dealing with psychiatric symptoms becomes overwhelming for people with SMI.

P5: *Most patients consume tobacco as a way to manage stress and relieve symptoms of psychiatric illness. So you see. Smoking is very, very common.*

Additionally, a small number of participants indicated that it was the most significant risk factor among people with SMI, which affects overall quality of life.

P3: *Smoking is widespread in people with mental illness, and it adversely affects its outcome and also impacts their quality of life.*

Participants confirmed the primary view of smoking as a coping mechanism, which manifests as either relieving psychiatric symptoms or managing stress in their lives. A significant number of people with mental health issues had behavioural risk factors like smoking and addressing concerns that significantly affected their quality of life.

### Theme 2: The Consequences of Smoking on Mental Health Issues

This theme explored the complex and bi-directional relationship between smoking and mental disorders. It came to an understanding that smoking and mental health issues were closely related and impacted the client’s way of dealing with the rest of the functional domains of their lives. People with SMI smoke often leads to decreased efficacy of psychotropic drugs and enormous burden in the form of financial constraints.

#### Efficacy of Psychometric Medication

Under this subtheme, participants emphasised the impact of smoking on medication by affecting the efficacy of psychotropic drugs along with increased enzymatic actions of the liver, which influenced their recovery.

1. *I think it’s well-known that smoking on a regular basis can reduce the efficacy of antipsychotic medication. So that is very important to note.*
2. *Smoking initiates problems in the metabolism of the drug so that the way patients get poorer responses with psychotropics prescribed by us.*

On a similar note, other participants conveyed that smoking impacted the client’s recovery and rehabilitation.

P9: *Smoking unquestionably influences the recovery process, affecting the recovery process; there’s no doubt about that.*

#### Smoking is a Costly Affair

Another subtheme revealed that the reason for non-adherence to medication prescribed to relieve symptoms of mental health issues was financial scarcity. More precisely, the participants conveyed that people with SMI opted to spend their money on buying cigarettes as compared to prescribed medicines. Owing to the limited finances and considering the cost of cigarettes daily, most of the participants agreed that patients usually attempted to replace psychotropic medicines with cigarettes, upholding their desire to buy cigarettes.

P2: *Smoking cigarettes is a costly affair. This financial restraint leads to the stopping of medicines rather than stopping cigarettes. And then there is a relapse of illness, and that vicious cycle keeps on repeating.*
P3: *Few of my patients I have advised them nicotine patches during the treatment, but the response was not that good. Surprisingly they say that it is costlier than the smoke itself and it does not reduce their craving.*

Smoking accelerates the metabolism of prescribed drugs among people with SMI, and this increased metabolism of psychotropic drugs contributes to the need for higher dosages to attain therapeutic efficacy, resulting in poor compliance. Moreover, there was financial restraint among people with SMI, either due to unemployment or dependency on family members, which led to choosing cigarettes over-prescribed medicines.

### Theme 3: Smoking Cessation: Patient-Related Factors

This theme highlights psychiatrists’ viewpoints on some of the factors that contribute to smoking cessation due to patients’ influence. An effective smoking cessation demands a collaborative approach that respects the patient’s readiness to adhere to cessation while integrating psychiatrists’ considerations into the cessation strategy. This theme included two factors: External and internal. Lack of peer and family support, along with the easy availability of cigarettes, were classified as external factors, while lack of motivation, awareness, and cognitive enhancement belong to internal factors.

#### External Factors

Psychiatrists highlighted that the significant aspect of smoking cessation was the lack of support from peers and family members. Participants confirmed that patients usually indulge in smoking to fulfil their desire to be accepted in their peer group, and such social surroundings posed additional barriers towards cessation. Another thing was that the acceptance of smoking among family members further hinders cessation. Moreover, many participants agreed that the easy availability of cigarettes within a fraction of a minute (Either from surroundings or peers) made it difficult to control the cravings among people with SMI.

P3: *Majorly the peer group or the easy availability of these substances like cigarettes makes them more indulged in smoking, which actually drives them away from cessation.*
P5: *Another strong factor is peer influence. They find a friend where they can sit and smoke and enjoy smoking with their peers.*

Participants also emphasised and acknowledged the easy availability of cigarettes or “biddi” in the nearby surroundings that hinder cessation.

P9: *Patients who are not working and who are dependent on family members also get access to these products and continue to smoke.*

This theme resonated with the shared experiences of participants in terms of lack of peer and family support. Psychiatrists expressed that family members often rationalised continued smoking for perceived calming effects.

P6: *Everybody’s happy when the doctor suggests or reduces and stops tobacco. Lots of time their was resistance from the family conveying to continue smoking to keep their patient calm and relaxed.*
P4: *Most of the time, they are not very resistant to smoking because they don’t agree with that. They are like it. Just leave it. Don’t talk about this.*

Family support was crucial in tobacco cessation; however, at times, resistance from family members with the rationale that quitting disturbs the client’s calmness. Getting such affirmations from family members eventually impacted the cessation outcome. Peer support could work as a motivator to quit smoking, but the availability of cigarettes makes it harder to obtain it.

#### Internal Factors

Several participants agreed that patients’ lack of motivation to quit smoking was the primary opposition encountered in clinical settings. Substituting smoking for calming effects becomes one of the rationales for continued smoking among people with SMI. Many of the participants expressed that smokers enjoy the habit of smoking and generally dismiss health concerns.

1. *They are poorly motivated; even though they initiate smoking cessation, they may not be able to go through it.*
2. *For surely, the patient’s motivation is always on the low side whenever we talk about smoking.*

Another common aspect was that a lack of awareness about the health impacts of smoking among people with SMI hinders cessation. The psychiatrist conveyed that smokers with SMI generally overlooked the consequences of smoking on their health and recovery and were more focused on resolving psychiatric symptoms.

P8: *The patient generally comes up by saying that his problem is the current symptoms of schizophrenia. I’m not interested in giving up smoking, so you see, they lack insight and awareness about the complications of tobacco.*

Another participant also shared a similar view:

P10: *The patient wants just a focused treatment of their mental illness. it looks like smoking treatment is not their concern.*

Participants conveyed that their clients often normalised smoking, with a justification that smoking was less harmful and helped them to accomplish cognitive tasks.

P4: *You can see the notions that they add to this. I did smoke that day and I was able to concentrate well on my studies. I smoked the other day, and I was able to perform my sexual activity better.*

Again, another participant also stated,

P6: *It is also true that patients lack insight and generally lack awareness and knowledge on ways to avoid smoking, especially when family members or any relatives smoke near them, which eventually sustains their craving for tobacco.*

It was insightful to discover that psychiatrists uncovered their observed thoughts about the lack of motivation and awareness among people with SMI. These factors act as potential barriers to engaging in focused cessation talk, which resulted in continued smoking among this vulnerable group.

### Theme 4: Smoking Cessation: Psychiatrist-Related Factor

This theme highlighted the factors related to psychiatrists regarding smoking cessation support provided to people with SMI. Almost all psychiatrists agreed that there was limited time in the context of a discussion on cessation, considering other priorities. There were consistently competing priorities with mental health issues like depression and aggression over smoking. Another common imperfection was the lack of adequate training among practitioners, which eventually hampered cessation.

#### Lack of Time

Within this subtheme, participants stated that time constraints in busy clinics or hospital settings usually limit in-depth cessation discussions. Even though some psychiatrists want to spend extra time, they have constraints in terms of the working window. These time constraints lead to missed opportunities for screening and comprehensive interventions.

P2: *It’s just a matter of the busy schedule and the time that some might not be focusing on cessation.*

Other participants shared similar views.

P6: *Time is always the barrier because I mean, of course, my clients would like to spend two hours with me. But as far as I’m concerned. If I have those hours for my general OPD, and I try to see only a set number of patients.*

According to participants, it was indeed the time constraint that they couldn’t stretch the conversation on smoking screening and cessation during patient consultation. In mental health care settings, discussion predominantly focussed on psychiatric symptoms, which was a priority at that point in time.

#### Priority of Care

Under this subtheme, participants expressed that respecting the complexity of managing people with SMI required prioritising mental health concerns over smoking. Smoking comes to the backseat due to other emergency clinical priorities related to the patient’s psychiatric symptoms.

1. *I think we psychiatrists are so focused on treating the bigger symptoms like the hallucination, the aggression, the other things. So, we don’t really treat smoking as such.*

Another participant also had similar views,

P10: *Another concern is prioritising smoking cessation in their care plan. Mental health issues often take priority, with limited space for discussions on smoking cessation. As it all depends on the psychiatrist’s perspective also, as I know some of the fellows who consider smoking causes minimal damage to the body.*

Furthermore, it became mandated to count smoking as a big problem, considering the increasing prevalence of the same. Nevertheless, for most psychiatrist, smoking has still not been ringing a bell in their heads.

P8: *As clinicians, some are not even considering smoking as a problem. So, there was a lack of flagging at the first or subsequent session, which is one of the hindrances to smoking cessation.*

Very few participants agreed that smoking needs to be a considerable concern as compared to other substance abuse. The mortality rate associated with smoking-related illnesses far exceeds that of other substances.

P3: *Like we give time to the alcohol addicted, we also don't give much time to smoking. It is sad, but we are not that serious about our approach.*

P9: *Apart from treating big symptoms, we, I think, are not much bothered about the patients being smokers. We are usually worried about alcohol or other drug abuse, but also I have generally seen that fellow psychiatrists didn’t consider it as a problem.*

Prioritisation was necessary, and the same should have been applied to smoking cessation. While other substance addictions were significant, smoking had greater harm to health and contributed to premature mortality.

#### Lack of Knowledge

This subtheme emphasised the lack of knowledge and training, especially at the postgraduate level in medical education. Participants stressed that inadequacy of training hampered cessation.

1. *I believe smoking cessation is not taught in postgraduate courses, so it is, you know unless doctors who want to take it up must undergo new learning. So now, doctors are not trained either.*

While one participant denied the above fact, conveying that psychiatrists were well-equipped in terms of cessation support.

P6: *I think psychiatrists are well-trained. Whether they are, you know, already while they are getting trained in institutes, but we’re also very, very good about upskilling ourselves otherwise. So, like I was saying, most psychiatrists are competent.*

Many participants pointed out the lack of comprehensive knowledge and training in tobacco cessation during postgraduate medical education. It not only acts as a significant barrier to assisting people with SMI but also lowers the confidence to deal with this issue. Enhancing cessation training among psychiatrists could equip them with the desired skills for practising screening and assessment in routine activities.

#### Patient Initiative/ Accountability

This subtheme elucidates those participants shared the agreement that if people with SMI sought to quit smoking and came to discuss cessation, then psychiatrists preferred to discuss cessation. It was practical to continue talking about cessation once the client was ready to engage in a conversation about quitting. The perceived benefits of quitting, combined with the motivation to sustain abstinence, made quitting appealing.

P3: *One of the things that we do not try much. We have accepted that they are going to smoke. And even I am going to break my head over it again multiple times, so most of us have accepted that. Yeah, it’s until the patient wants to stop smoking.*

Another participant also shared that,

P5: *We, as psychiatrists, accepted the lack of the patient’s motivation for cessation and understood that we were flogging the tired horses, not going to leave. He has made up his mind. And then suppose we insist too much, then he’ll start lying, so unless the patient himself comes and says that I want to stop smoking.*

Similarly, another participant expressed that,

P9: *If the patient comes to us and wants to explore options for smoking cessation, we are more than happy to help.*

Participants conveyed that they wanted to help the client until and unless the client himself wanted to take the first step and work on ending smoking. However, they agreed to provide treatment support once the client was convinced to take that challenge.

### Theme 5: Catching the Craving: Applying the 5A

This theme emerged from the screening and discussion around smoking through the World Health Organisation’s (WHO) 5A scale. Varied participants agreed that they use this standardised scale to engage in a conversation about assessing smoking patterns and advising to abstain from it.

#### Consistently Engaging in 3As (Asking, Advising, and Assessing)

This subtheme discussed how participants are addressing concerns over smoking on the WHO 5A scale, which involves asking, advising, and assessing. Most of them agreed that employing the first three components of 5A (Ask, assess, and advise) was characteristically part of the routine process in outpatient department (OPD) conversations.

P9: *Truly speaking, I think asking, advising, and assessing is easy on our part, as these are on the psychiatrist’s part. We utilise these components during the first patient visit.*
P1: *I think asking and assessing are routine. Even advice is regularly given at the end of the conversation.*

Another participant also mentioned.

P10: *Regarding the WHO tool, Asking is the most used method of discussing with patients, so I believe it is the easiest one. Every psychiatrist does it while taking a history or discussing priority complaints. Advice is also one part that we usually do daily and always include while providing care to patients.*

Indeed, participants conveyed that the WHO 5A tool was most utilised when asking their clients about smoking. As for assessing and advising components, participants agreed that it was regularly executed while providing care to patients.

#### Challenges Associated with Other 2As (Assisting and Arranging)

Participants expressed their constraints in terms of utilising assistance and arranging from the WHO 5A scale due to limited available services and high failure rates. Taking into consideration complex mental health conditions and the low motivation of people with SMI, it becomes hard to arrange and assist.

P3: *Certainly, the next two steps requiring additional resources and equipment, like assisting or arranging, may be the hardest ones. There is limited availability of material, especially time, from psychologists for every patient to discuss their issues.*

Again, similar views were shared by another participant.

P4: *I can say assisting and arranging is a bit difficult for patients. Though I tried my best, I know sometimes. All of our efforts are not enough. Without the support of the government or organisations in terms of drugs or counsellors or any dedicated employees for this problem, it becomes difficult for psychiatrists to handle it all alone.*

Participants expressed a sense of despair in terms of assisting and arranging services related to smoking cessation among people with SMI. The inability to arrange counselling services and the need for other resources (drugs or pamphlets) made it harder to do in clinical settings. Moreover, it was also difficult for the patients, as they needed to pay extra money for the utilisation of these services.

## Experience and Challenges Around “Cessation Talk”

This theme revealed the experiences of participants regarding initiation and discussion on cessation talk among people with SMI. Many of them described it as confronting a situation where they handled primary (psychiatric) symptoms first and later proceeded toward smoking cessation. Initial opposition from patients was a familiar gesture, which psychiatrists dealt with by emphasising the physical and psychological effects of smoking on overall health.

### Initiating Cessation Talk

Within this subtheme, participants communicated that patient initially resisted and preferred to focus solely on their primary illness. Later, once symptoms stabilise, discussions about cessation could be introduced.

P3: *Initially, we didn’t discuss their smoking as we know once the discussion goes toward smoking, the patient tries to avoid further conversation.*
P6: *When the symptoms are better, they’re doing well, they’re maintained on medications, and their functioning has improved a little bit. We have then those conversations. Then we know that the motivation isn’t that great, but we make a point, and then we ask again next month.*

Similar views were shared by other participants, too.

P10: *We usually start by making them aware of the benefits of quitting smoking and go along with their pace and varied therapies and drugs.*

P9: *In a typical scenario, after a full assessment and assessing their motivation level and severity of smoking, we proceed to guide them through steps for cessation; we generally start with slow reduction along with drug support and with minimal disturbances in their psychiatric treatment.*

Smoking cessation talks were deferred initially to maintain patient engagement. Afterwards, focusing on addressing psychiatric issues like depression and anxiety came into play, where psychiatrists gradually integrated smoking cessation efforts as psychiatric symptoms stabilised.

### Lack of Readiness

The participants under the subtheme prioritise treating the primary condition like schizophrenia as most of the clients were not ready to accept or initiate cessation. Additionally, patients didn’t even consider tobacco as harmful as other substances like alcohol.

P3: *Truly speaking, they don’t give it much importance, and if we keep on asking them many times, they get irritated by saying what is the big deal in smoking.*

Other participants also shared their views below.

P5: *The majority, as I told you, are not willing to stop, and they are not happy to abstain from their smoking activities. So they are absolutely fine, and whatever other disorder, depression, bipolar, or alcohol, they are willing to take the treatment for the same.*

Participants firmly believed that psychiatric conditions made it challenging to consider smoking over mental health issues. It was essential to understand that without the acceptance or readiness of the client, it has been difficult to deal with cessation.

### Rolling with Resistance

Under this subtheme, participants expressed their adaptation with resistance while contemplating the cessation. Many of the participants agreed that the patients are not motivated to abstain from smoking, and challenging them yields no benefits.

P9: *I can understand that patients usually do not consider it as something terrible to their health and are not interested in talking or listening to tobacco cessation, so we are helpless and stop the discussion. You know otherwise, patients start to have resistance and usually don’t take treatment seriously.*
P2: *We focus on treating the present condition, like treating schizophrenia; in the next couple of visits, we will target cessation because that is going to affect my treatment. It is their habit, so we can’t test the challenge directly; if we do so, he will not come to me for a follow-up of his psychiatric.*

Again, another participant shared similar views.

P4: *The majority of them are in the pre-contemplation stage, wherein they disagree that they have a problem.*

Participants concluded that people with SMI were often in the pre-contemplation stage regarding smoking, not recognising it as a problem. However, cessation was crucial due to its impact on medication efficacy and health outcomes. Patients often downplay smoking despite its severe health risks.

### Highlighted Physical Health Outcomes

Within this subtheme, participants underlined that when patients showed interest in quitting smoking, it became necessary to relate to their physical condition to make them understand the whole context. Participants anticipated their patients’ willingness and later engaged with them for cessation support.

P9: *You see, most of our patients are not eager to quit smoking unless they face serious health issues like a stroke or a heart attack caused by smoking. That is when they become more motivated to quit to treat their physical illness, which they fear dying and asking to resolve the issue of smoking. That is when we start talking about stopping smoking.*

Another participant stated.

P8: *As I said, in the first few sessions, we will be able to get them to engage in conversations about tobacco cessation. However, I will flag it for them that this is an issue, and I want you to think about whether you want to quit or not. My approach would be that I am not going to push for it, at least for the next few visits.*

It was evident that instead of concentrating merely on verbal discussion related to cessation, the deteriorating effect on physical health was far more effective in convincing people with SMI regarding cessation support. Every doctor, including cardiologists, should advocate cessation, as many patients quit after significant health events like heart attacks or strokes.

### Theme 7: Decoding Demographics and Recommendations

Interestingly, this theme stressed that the male gender had the most common pattern of smoking. Participants discussed the high prevalence of smoking among males who were diagnosed with SMI, especially in rural contexts.

P2: *We have seen mainly in the male gender, those who smoke a lot in early adulthood or late adolescence and the rural population.*
P7: *We saw the majority as male gender. That is there, at least here in India. We don’t get to see females who smoke. I have also observed that older people consider it a part of their regular activities or typical days. They aren’t much bothered by it.*

Similarly, another psychiatrist expounded.

P10: *The major portion of the male population smokes the most. Though smokeless tobacco is also a concern, smoking is the most common one.*

In terms of future recommendations, participants devised fascinating yet practical solutions for dealing with effective cessation. Some of the participants elaborated on classical ideas about teaching behavioural and cognitive tools to help patients manage emotions and anxiety. Few agreed that smoking cessation was a shared responsibility across medical specialties.

P 9: *As I told you many times, our patients are using cigarettes to regulate their emotions or to take care of their anxiety. So if we are able to teach them those tools on how to manage your emotions or your behaviour without going to smoking, you know, like breathing exercises, distracting yourself, etc.*

Similar views were shared by other participants too.

P7: *Everyone should be trained. It is not a psychiatrist's job alone, and many patients go to pulmonologists or physicians.*

Another participant suggested a comprehension of cessation in the patient-provider relationship, which was crucial for achieving effective cessation.

P3: *My personal experience concludes that we cannot tackle this problem thoroughly until there is a both-sided demand for smoking cessation. First, each patient should be asked about this as part of our approach to management. Second, and most important, is counselling or psychoeducation and family support.*

Participants expressed that advocating for smoking cessation should be a standard component of psychiatric rehabilitation programs. Mutual motivation from both people with SMI and psychiatrists is essential to effectively address this issue. The discussion highlighted that dedicating time for these conversations, despite perceived constraints, could lead to satisfactory cessation support.

## Discussion

This study identified seven themes related to psychiatrists’ views on smoking cessation among people with SMI. Furthermore, fourteen subthemes were devised, focusing on the scope that highlighted varied factors that impact psychiatrists’ outlooks on smoking cessation among people with SMI. This research found that participants perceived that patients use smoking as a way of relieving symptoms or improving mood, which is another perceived barrier endorsed by participants that hinders cessation (Rogers et al., 2018). In the current study, participants expressed their views in terms of the limited feasibility of employing time on smoking cessation when patients were faced with other psychiatric symptoms. Similar themes emerged from qualitative studies (Kerr et al., 2013; Rogers et al., 2018) that adhered to competing priorities and patient resistance in terms of smoking cessation. Moreover, patient resistance was the main reason for providers to abstain from delivering cessation conversations.

The findings of this study provide qualitative results, stipulating robust evidence of participants’ views related to time constraints while providing OPD services to people with SMI. Likewise, Malte et al. (2013) testified that time constraints were the most common barrier reported by practitioners. Findings from another research conveyed those participants faced difficulties in cessation due to patient resistance to quitting or treatment, which acted as a substantial obstacle that was reported by other studies (Brown et al., 2015; Wilson et al., 2016), confirming their perception that people with SMI were not interested in quitting. Another study supports that it was very challenging to engage people with SMI (schizophrenia) in cessation talk (Rogers et al., 2018). Moreover, a recent study (Papadakis et al., 2020) conveyed that even patients with a low level of readiness require support from their physician for cessation.

Patient initiative in exploring treatment options for smoking cessation in mental health care settings would not only lower dependence on providers’ initiative but also strengthen implementation of tobacco treatment (Rogers et al., 2018). Participants came up with another subtheme, where cessation was shared responsibility in terms of other providers as well as people with SMI. A similar idea was supported by other studies (Rogers et al., 2018; van Rossem et al., 2015), stating that smoking was a shared responsibility between mental health and primary care. Another subtheme highlighted by participants was limited training in terms of smoking cessation, which was aligned with another quantitative (Okoli et al., 2017) and qualitative study (Rogers et al., 2018) affirming this notion. This limited training contributes to the insight that smoking cessation was not one’s responsibility but required collaborative efforts.

The present findings were focused on the lack of training among some of the participants, which was a mandate for smoking cessation from the psychiatrist’s perceptions (Glover et al., 2014). Another qualitative study revealed that few mental health professionals had undertaken training in general addiction or, more specifically, in alcohol addiction, but smoking cessation training was overlooked (Kerr et al., 2013). In the current discussion with participants, the lack of training can be fulfilled with efforts from the organisation, especially on smoking cessation. This training can enhance staff interest and boost positive attitudes toward cessation in their healthcare settings. Many of the participants reported that tobacco was a low priority for their patients (Ziedonis et al., 2006) as patients preferred to have conversations addressing their’ agendas relating to the current issues before discussing smoking.

Patient’s interest and engagement in cessation could be encouraged once their provider addresses those barriers, which eventually assisted in clearing out the perceived blockades for successful cessation. Some participants confirmed that initiating cessation talk in OPD consultations impacted their doctor-patient relationship as practitioners attempted to preserve these relationships and prevent negative client reactions (Kerr et al., 2013). Psychiatrists need to be aware of their beliefs about their patient smoking behaviour. It was acknowledged to have a genuine concern regarding smoking and its cessation, yet this anxiety about damaging the relationship could negatively affect efforts and interventions for quitting smoking. Another concern raised by psychiatrists was fear that smoking cessation impacted mental health recovery (van Rossem et al., 2015; Wye et al., 2010), as it was well evident that nicotine impacted the metabolism of psychotropic drugs, which resulted in reduced efficacy of the same drug. Our findings provided inferences for better-developing strategies or policies that support smoking cessation, contemplating psychiatrists’ perspectives. This must involve not only adhering to one theme or viewpoint but conjoining all these themes, which could result in the remarkable and systematic implementation of tobacco treatment for people with SMI.

## Limitations

In this study, discussions with psychiatrists about smoking cessation were focused on people with SMI during their OPD consultations only, highlighting a need to integrate routine screening for smoking cessation with mental health treatment. Another constraint was limited information on the smoking status of psychiatrists, which might have impacted their views towards treating patients with tobacco use. Despite these limitations, this study remains a valuable contribution for psychiatrists in Indian settings, shedding light on their perspective related to smoking cessation. This information would be utilised in tailoring workshops and seminars focussed on overcoming barriers related to cessation.

## Implications

An important implication of this study is the need to bring the issue of smoking cessation to psychiatrists’ attention and encourage discussions during patient visits. This necessitates specialised training and support for psychiatrists to build or enhance their ability to provide adequate smoking cessation assistance to people with SMI. Such training will also help psychiatrists deliver a tailored approach, drawing on findings from this study. Moreover, the study emphasises the integration of smoking cessation into the standard management practices for people with SMI, which can lead to significantly improved health outcomes. Psychiatrists need to adopt a proactive role in addressing smoking habits alongside comprehensive care, ultimately contributing to a reduction in smoking-related diseases and mortality. Another critical aspect is the lack of time. Even though psychiatrists may have the necessary skills and willingness to assist patients in quitting smoking, they might be hindered by time constraints. The demanding nature of psychiatric practice and the necessity to prioritise acute mental health concerns often leave little room for addressing tobacco use. This calls for system-level changes in operational policies, such as integrating smoking cessation into routine psychiatric care, allocating dedicated time for preventive interventions, and employing multidisciplinary teams to support smoking cessation initiatives.

## Conclusion

Examining psychiatrists’ views on smoking cessation in people with SMI is crucial for achieving lasting health benefits. In this study, psychiatrists recognised the rationale and reason for smoking cessation among people with SMI, either smoking as a coping mechanism or relieving stress. Time constraints, task priority, and attempts to sustain good therapeutic relationships with patients were some of the emphasised themes by psychiatrists while considering smoking cessation. These findings provide valuable insight for understanding psychiatrists’ views while attempting to incorporate cessation in clinical settings. Integrating cessation talk with primary consultation can provide valuable insights for catering to future interventions for cessation.
